# The p53 Pathway in Glioblastoma

**DOI:** 10.3390/cancers10090297

**Published:** 2018-09-01

**Authors:** Ying Zhang, Collin Dube, Myron Gibert, Nichola Cruickshanks, Baomin Wang, Maeve Coughlan, Yanzhi Yang, Initha Setiady, Ciana Deveau, Karim Saoud, Cassandra Grello, Madison Oxford, Fang Yuan, Roger Abounader

**Affiliations:** 1 Department of Microbiology, Immunology & Cancer Biology, University of Virginia, Charlottesville, VA 22908, USA; yz5h@virginia.edu (Y.Z.); cjd7ua@virginia.edu (C.D.); mkg7x@virginia.edu (M.G.J.); nac5t@virginia.edu (N.C.); baomin@virginia.edu (B.W.); cough25m@mtholyoke.edu (M.C.); yxy627@case.edu (Y.Y.); is7fs@virginia.edu (I.S.); ced5vr@virginia.edu (C.D.); kgs3fd@virginia.edu (K.S.); cmg2dk@virginia.edu (C.G.); mao5bd@virginia.edu (M.O.); fy5dm@virginia.edu (F.Y.); 2 Department of Neurology, University of Virginia, Charlottesville, VA 22908, USA; 3 The Cancer Center, University of Virginia, Charlottesville, VA 22908, USA

**Keywords:** glioblastoma, wild type p53, mutant p53, gain-of-function

## Abstract

The tumor suppressor and transcription factor p53 plays critical roles in tumor prevention by orchestrating a wide variety of cellular responses, including damaged cell apoptosis, maintenance of genomic stability, inhibition of angiogenesis, and regulation of cell metabolism and tumor microenvironment. *TP53* is one of the most commonly deregulated genes in cancer. The p53-ARF-MDM2 pathway is deregulated in 84% of glioblastoma (GBM) patients and 94% of GBM cell lines. Deregulated p53 pathway components have been implicated in GBM cell invasion, migration, proliferation, evasion of apoptosis, and cancer cell stemness. These pathway components are also regulated by various microRNAs and long non-coding RNAs. *TP53* mutations in GBM are mostly point mutations that lead to a high expression of a gain of function (GOF) oncogenic variants of the p53 protein. These relatively understudied GOF p53 mutants promote GBM malignancy, possibly by acting as transcription factors on a set of genes other than those regulated by wild type p53. Their expression correlates with worse prognosis, highlighting their potential importance as markers and targets for GBM therapy. Understanding mutant p53 functions led to the development of novel approaches to restore p53 activity or promote mutant p53 degradation for future GBM therapies.

## 1. Introduction

The *TP53* gene is located on human chromosome 17p13.1. It encodes the p53 protein that consists of 393 amino acids (https://genome.ucsc.edu/). Functional p53 is a homotetramer that adapts a dimer-of-dimers topology [1,2]. P53 acts as a transcription factor that is composed of distinct domains. The nucleotide-binding domain interacts with the consensus DNA sequence for p53 [3,4]. The other domains communicate with diverse regulatory pathways and further regulate transcriptional activity. Under normal conditions, p53 activity is low and checked by MDM2 and MDM4 (MDMX) through ubiquitination and degradation [5]. The interaction between p53 and MDM2 is disrupted in response to stress signals such as DNA damage, leading to p53 induction.

P53 plays a central role in maintaining cellular homeostasis and is frequently deregulated in cancer. The protein is positioned at the center of a regulatory network that controls cell proliferation, survival, genome integrity and other functions. Functioning as a transcriptional regulator, p53 integrates stress signals and promotes cell cycle arrest, senescence and apoptosis to prevent damaged cells from propagation [6]. P53 is thus named the “Guardian of the Genome” [7]. Recent research advances on p53 have revealed additional roles beyond gene transcription regulation and protection of genome integrity. Among other, evidence has shown that p53 is an important regulator of cellular metabolism, stemness, autophagy, invasion, metastasis, microenvironment, and immunity [6,8,9].

Glioblastoma (GBM; grade IV glioma) is the most common and most deadly primary malignant brain tumor in humans. The survival of GBM patients is only about three months without prompt treatment [10]. Current GBM therapies include surgery, radiation therapy, and chemotherapy. However, even with these therapies, the average survival of GBM patients after diagnosis is only ~15 months [11]. Gliomas are classified into four grades based on the World Health Organization. Glioblastoma patients typically present with de novo grade IV lesions delineating primary glioblastoma (85–90%) [12]. Low-grade gliomas can progress into grade IV lesions delineating secondary glioblastoma [12]. One of the key molecular differences between primary and secondary glioblastoma is the presence of isocitrate dehydrogenase-1 (IDH1) mutations which are found in approximately 75% of secondary GBM but are rare in primary GBM [13]. The mutational landscape of primary and secondary GBM also differs with regards to *TP53* as well. The latest iteration of The Cancer Genome Atlas (TCGA) GBM project shows that the p53 pathway (including CDKN2A, MDM2 and TP53) was deregulated in ~85% of tumors [14]. Upon closer analysis, the *TP53* gene was mutated in ~28% of tumors with lower grade gliomas exhibiting >90% *TP53* mutations in co-occurrence with IDH1 mutations [14]. Primary GBM can be molecularly subdivided into different subtypes based on their differing mutational patterns: proneural, mesenchymal, neural, and classical [15]. The prevalence of p53 mutations differs in the different GBM molecular subtypes (proneural, mesenchymal, neural and classical) (54%, 32%, 21% and 0%, respectively) [15]. TP53 expression has been compared in newly diagnosed patients and recurrent tumors with inconsistent results as to whether expression is altered upon recurrence [16,17,18]. In this review, we summarize the current understanding of p53 in GBM.

## 2. P53 Role and Deregulation in GBM 

### 2.1. The p53 Pathway is Frequently Deregulated in GBM

**Figure 1:**
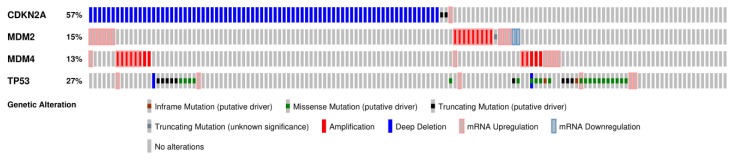
The p53 pathway is highly deregulated in GBM (adapted from cBioportal) [22,23]. The most common mutations of the p53 pathway are missense mutations in *TP53,* deletions of *CDKN2A/ARF*, and/or amplifications of *MDM2* and *MDM4*. These mutations often lead to diminished tumor suppressor activity.

*TP53* is one of the most commonly deregulated genes in cancer. It is deregulated in as few as 10% of cases (hematopoietic malignancies) or in as many as 100% of cases (high-grade serous carcinoma of the ovary) [19]. The p53 pathway is also frequently deregulated in GBM. Although little is known about the mechanisms underlying GBM initiation, modeling through manipulation of mice genetics suggests that pathways involved in growth factor-induced signal transduction and processes that control cell cycle progression may be implicated. The ARF-MDM2-p53 pathway is one such pathway, and is deregulated in 237/281 (84%) of GBM according to The Cancer Genome Atlas (TCGA) (TCGA, 2013) and in up to 94.1% of GBM cell lines [20,21]. *TP53* is deregulated in 22% of GBMs, while ARF, MDM2, and MDM4 are deregulated in 61%, 10%, and 10% of GBMs, respectively (Figure 1) [22,23].

### 2.2. P53 Is Implicated in GBM Progression.

**Figure 2:**
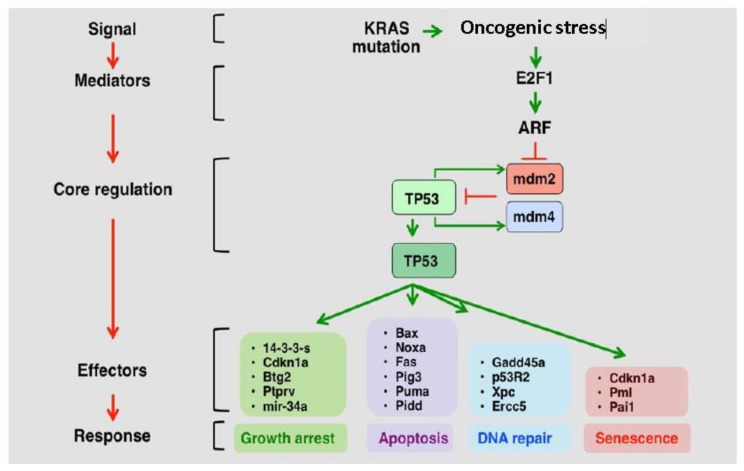
The p53-ARF-MDM2/4 pathway (adapted from the p53 website) [24]. In response to hyperproliferative stress and/or DNA damage, pathway mediators, such as ARF, are activated. MDM2 and MDM4 mark p53 for degradation, and are subsequently degraded when upstream mediators are activated. This releases p53 from degradation, and leads to increased cell cycle arrest, apoptosis, DNA Repair, and cellular senescence.

P53 exerts tumor suppressor activity primarily by altering the expression of numerous genes involved in cell cycle arrest, apoptosis, stem-cell differentiation (Figure 2) [24] and cellular senescence [25,26]. It is commonly activated in response to DNA damage, genotoxicity, oncogene activation, aberrant growth signals, and hypoxia, all of which are events that can be encountered during carcinogenesis [27]. Although *TP53* mutations are oncogenic in GBM, other genes, such as PTEN must also be mutated to drive GBM progression [28].

The mutational status of *TP53* is associated with GBM progression [29] and p53 inactivation is correlated with a more invasive [30], less apoptotic [31], more proliferative [27], and more stem-like [28] phenotype. GBM cell lines possessing inactivated mutant p53 (mut-p53) are more resistant to DNA-damaging therapeutic drugs, such as cisplatin [31]. Although mut-p53 has been correlated to poorer prognosis in breast and other cancers [32], the mutational status of *TP53* and its various pathway elements (ARF-MDM2/4) has not been correlated with survival in GBM, despite the high mutation frequency [27,32,33,34].

### 2.3. CDKN2A/ARF Is the Most Commonly Deregulated Component of the p53 Pathway in GBM

The most frequently deregulated component of the p53 pathway is a homozygous deletion of the CDKN2A/ARF locus, which occurs in ~60% of all GBM cases (Figure 1) [22,23]. These deletions are more commonly found in younger GBM patients [35]. ARF exerts tumor suppressor activity through its ability to promote MDM2 degradation, thus preventing the degradation of p53 tumor suppressor activity (Figure 2) [24]. In addition, ARF serves as a mediator of antiangiogenic effects by upregulating expression of tissue inhibitor of metalloproteinase-3 (TIMP3). ARF induction of TIMP3 has been shown to reduce GBM cell migration [36]. ARF deletion is correlated with overexpression of tectonic family member 1 (TCTN1), a protein involved in a diverse range of cellular processes, including promotion of GBM cell proliferation. The deletion of CDKN2A/ARF has also been correlated with GBM tumors displaying prominent adipocytic-like tumor cell differentiation. This produces a phenotype that is distinct from classical GBMs [37,38]. CDKN2A/ARF and *TP53* are rarely co-deregulated, suggesting redundancy in the effects of their loss on GBM malignancy [39,40]. CDKN2A/ARF loss has not been shown to correlate with GBM prognosis and/or survival [27,32,33,34].

### 2.4. MDM2 and MDM4 Are Amplified in GBM and Negatively Regulate p53

Within the p53 pathway, MDM2 and MDM4 act as oncogenic inhibitors of p53’s tumor suppressive activity. MDM2 targets p53 for degradation, acting as a negative regulator [27,41]. MDM2 transcription is induced by p53, creating a negative feedback loop regulating the activity of p53 and the expression of MDM2 [41]. Amplification of MDM2 and MDM4 can inactivate p53, leading to loss of various tumor suppressor functions including growth arrest, apoptosis, DNA repair, and senescence [27,41]. An assessment of chromosomal imbalance using array comparative genome hybridization and WGA-DNA from two to five separate tumor areas of 14 GBMs demonstrated that there was a list of genetic alterations that were common to all tumor areas, including amplifications in 1q32.1 and 12q15, which contain MDM4 and MDM2 [42]. Furthermore, digital karyotyping, used to search for genome-wide DNA copy alterations in eight GBM cell lines and one bulk tumor sample showed that amplifications were also found in 1q32.1 and 12q15 [43]. MDM2 amplifications are more common in classic GBMs when compared to GBMs with astrocytic and oligodendroglial differentiated areas [38]. MDM2 amplifications and *TP53* mutations are mutually exclusive [38]. MDM2 amplifications occur in ~6.7% of all GBM cell lines, and only occur in cells that possess wild type p53 (wt-p53) [44]. Mut-p53 can be degraded by MDM2, but cannot transactivate MDM2, thus allowing the mut-p53 to escape degradation and exert mutational gain function effects [27]. According to the TCGA, MDM2 and MDM4 amplifications are found in 14% and 7% of GBMs, respectively [41]. When combined with temozolomide, use of the MDM2 inhibitor, nutlin3a, correlated with activation of the p53 pathway, downregulation of DNA repair proteins, persistence of DNA damage, and decreased cellular invasion [45]. MDM2 amplifications have not been shown to correlate with GBM survival and/or prognosis [27,32,33,34]. 

### 2.5. The p53 Pathway Is Regulated by Various Non-Coding Elements in GBM

While most cancer research has focused on protein coding genes, over 98% of the human genome that is transcribed consists of various classes of non-coding transcripts. Of these, microRNAs (miRNAs) [46,47] and long non-coding RNAs (lncRNAs) [48] are important regulators of cancer biology. miRNAs are a class of non-coding RNAs that are 21–25 nucleotides in length [49,50]. miRNAs serve as critical regulators of gene expression by targeting and inhibiting one or more messenger RNA (mRNA). As such, these molecules also regulate the p53 pathway in GBM. Mir-34a is as a transcriptional target of p53 and is commonly downregulated in GBM, and negatively affects GBM cell proliferation, invasion, cell survival, and cell cycle progression [51]. P53 is in turn regulated by miR-125b, an oncogenic miRNA that is upregulated in GBM and that inhibits cell proliferation and promotes evasion of apoptosis [52]. Mir-124 targets PPP1R13L, a p53 pathway inhibitor, and reduces GBM cell proliferation, cell cycle progression, and invasion. Mir-124 is commonly deregulated in GBM, and its downregulation is believed to promote GBM malignancy through elevated PPP1R13L [53].

In addition to p53, the other members of the p53-ARF-MDM2/4 pathway are also known to regulate and be regulated by miRNAs. The CDKN2A/ARF locus is frequently co-deleted with mir-491, which has been shown to reduce GBM cell invasion and proliferation. CDKN2A null mice display an exacerbated invasive phenotype when mir-491 is knocked down [54]. Mir-17 can repress MDM2 levels and reduce cell proliferation by protecting p53 from MDM2-mediated degradation [55]. Mir-217 is known to target and reduce expression of YWHAG, which is known to accelerate phosphorylation of MDM4 and increase degradation of p53.

Long non-coding RNAs (lncRNAs) are another class of non-coding RNAs that are greater than 200 nucleotides in length. They have diverse roles, including acting as proteins scaffolds, regulation of alternative splicing, association with chromatin remodeling complexes and association with enhancer function [56]. These understudied molecules are known to be deregulated and to serve diverse roles in cancer progression [48]. Two lncRNAs, H19 and UCA1, regulate GBM progression by interacting with mir-140 and mir-182, respectively. Mir-140 and mir-182 downregulate inhibitors of *TP53* leading to reduced cell proliferation and increased cell apoptosis, as per normal p53 function. H19 and UCA1 reduce the expression and activity of these miRNAs, and therefore possess p53-dependent oncogenic activity in GBM [57,58].

### 2.6. Gain-of-Function Mut-p53 in Cancer and GBM

**Figure 3:**
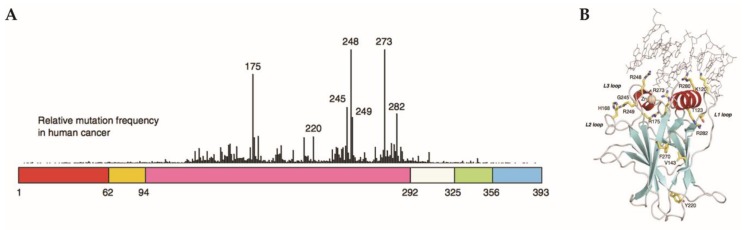
Most *TP53* mutations are in the DNA binding domain. (**A**) The schematic view of the domains of p53 protein. The protein has 393 residues with numerous different domains including the N-terminus (**red**), proline rich domain (**orange**), DNA binding domain (**purple**), tetramerization domain (**green**) and negative regulatory domain/C-terminus (**blue**). The peaks represent the frequency of mutations in cancer with the DNA binding domain containing the six hot spot mutations. (**B**) Ribbon diagram of DNA-bound p53 (PBD ID 2AHI). The residues highlighted in yellow represent residues that are hot spot mutations as well as other residues of interest. Modified and reprinted with permission from Springer Nature: [Springer Nature] [Oncogene] [112].

Unlike most tumor suppressors, *TP53* is rarely deleted in GBM. Most *TP53* alterations in GBM are missense mutations in the DNA binding domain (DBD), leading to inhibition of transcription factor activity (Figure 3). Importantly, mut-p53 protein is highly expressed in GBM and numerous studies have demonstrated that mut-p53 possesses oncogenic functions beyond those acquired through wt-p53 loss. These p53 mutants have been designated gain-of-function (GOF) mut-p53. GOF was first described by the demonstration that expression of mut-p53 in cell lines/mice lacking endogenous wt-p53 enhanced tumorigenic potential [59]. Subsequent knock-in studies have also demonstrated distinctive GOF activity associated with mut-p53 [60]. More than 25% of *TP53* missense mutations occur within 6 “hotspots” between exons 4–8: R273, R248 (class I, DNA contact mutations) R175, G245, R282, and R249 (class II, conformational mutations) (Figure 3) [61]. However, mutations in all *TP53* DBD codons have been reported in cancer [62]. 

Increased expression of GOF mut-p53 is due to the disruption of the MDM2/p53 negative feedback loop. Wt-p53 levels are regulated by the E3 ubiquitin ligase MDM2 [63]. MDM2 is a downstream target of wt-p53, forming a negative feedback loop, which is disrupted in mut-p53 [64]. Interestingly, while mut-p53 fails to transactivate MDM2, mut-p53 is still susceptible to MDM2-mediated ubiquitination. The disruption of this negative feedback loop provides a mechanism for mut-p53 accumulation [65]. Extrinsic factors, such as truncated MDM2 isoforms, have also been shown to enhance mut-p53 accumulation [66]. Furthermore, the stabilizing chaperone activity exhibited by Hsp90 that is overexpressed in many human tumors inhibits the proteasome-dependent degradation of mut-p53 [67]. 

GOF mut-p53 is associated with enhanced proliferation, migration, invasion and resistance to chemotherapy [59,68,69]. In addition, GOF mut-p53 induces an anomalous, carcinogenic metabolism (i.e., lipid metabolism and Warburg effect) and disturbed tissue architecture [70,71]. The amplified malignant properties displayed by GOF mut-p53 can be attributed to an acquisition of novel, aberrant transcriptional activity [72]. In contrast to wt-p53, GOF mut-p53 exhibits interactions with distinct transcription factors (e.g., NF-ɣ, SREBP, VDR, Sp1, ETS2, NFR2, p73, p63) that promote tumor initiation and progression [13,72,73,74,75,76,77]. 

Further sustaining its tumorigenic effect, mut-p53 can exert a dominant-negative regulation over wt-p53 through protein-protein interactions and the formation of oligomeric complexes [78,79]. P73 and p63, protein family members of p53, have been shown to recoup the tumor suppressive functions of p53 in mut-p53 absence. However, like wt-p53, this activity is inhibited by protein-protein interactions between mut-p53 and p63/p73. Moreover, mut-p53 inhibition of p63 and p73 promotes tumor cell invasion [80,81]. Mut-p53 further enhances proinvasive signaling through transcriptional upregulation of prosurvival receptor tyrosine kinases (RTKs) such as MET and EGFR, suppression of receptor targeting miRNAs, or by escalating receptor recycling [81].

As a result of disturbed regulatory checks and novel transcriptional activity, mut-p53 tends to localize and accumulate in the nucleus [73]. In contrast, wt-p53 exhibits a short protein half-life and is maintained at low intranuclear levels, only stabilizing in response to cellular stress. Wt-p53 stabilizes as a consequence of a multitude of post-translational modifications along ~60 residues [82]. The majority of these residues are not localized within the DBD, resulting in infrequent mutations. As such, these sites remain active in mut-*TP53* and the stress signals that stabilize wt-p53 also stabilize mut-p53 [83]. 

GOF mut-p53 is understudied in GBM. Most studies on p53 in GBM failed to distinguish between *TP53* deletion and GOF mutations [84,85]. TP53 mutations have been reported both in primary and secondary grade lesions (30% and 65%, respectively) [86,87]. Epidemiological studies demonstrate that “hot spot” codon mutations are common in GBM (specifically, 248 and 273), with higher rates in secondary GBM, suggesting that the acquisition of *TP53* mutations may differ between primary and secondary GBM [88,89]. It was suggested that *TP53* mutations in secondary GBM occur early on, whereas primary mutations occur later in consequence of heightened genomic instability. This implies that mut-p53 may be associated with the progression from low grade to high-grade lesions [84,86]. Differentiation between primary and secondary lesions is emphasized with regards to p53 subcellular localization. Primary de novo tumors demonstrated preferential p53 cytoplasmic localization in comparison to secondary GBM tumors, suggesting that delocalization may play a unique role in de novo lesions [84]. However, this study did not discern between wt-p53 and mut-p53, which reaffirms a necessity for reevaluating the influence of GOF mut-p53 in GBM progression. Previous studies have alluded to cytoplasmic p53 displaying non-transcriptional roles in promoting apoptosis and inhibiting autophagy [90]. Elucidating the presence of wt-p53 or GOF mut-p53 in the cytoplasm may provide more insight into the deregulation of p53 tumor suppressing activity in GBM lesions. 

The prevalence of *TP53* mutations also differs in the different GBM molecular subtypes (proneural, mesenchymal, neural and classical) (54, 32, 21 and 0%, respectively) [15]. As such, understanding the molecular signature of mut-p53 in different grades and subtypes of GBM may have clinical significance [91]. For example, upregulation of PDGFRA, platelet-derived growth factor receptor alpha, is a hallmark of the proneural subtype [15]. Aberrant upregulation of PDGFRA and loss of wt-p53 activity corresponded to more aggressive GBM tumors. Such findings warrant a reevaluation of the role of mut-p53 in the proneural subtype [92,93]. Furthermore, GOF mut-p53 has been shown to abnormally regulate a multitude of genes that play critical roles in GBM pathogenesis, but have not been analyzed in the context of GBM. This includes CD95/Fas/Apo1, PARP, PTEN, and more [63,94,95,96,97,98]. Also, the role of mut-p53 in proliferation has been examined with the R280T mutation along the DBD promoting proliferation through the GSK-3β/PTEN pathway in GBM cells [99].

GBM stem-like cells (GSC’s) contribute to GBM resistance to treatment and enhance the proliferative capacity of tumors. Wt-p53 has been suggested to influence stem cell migration along with differentiation, depending on cellular context [100]. Somatic niches maintain the quiescence of stem cells and disruption of this microenvironment may contribute to proliferation and malignancy. For example, neural stem cells (NSCs) lacking wt-p53 prematurely exit from the neurogenic niche and are theorized to contribute to tissue invasion and the development of GBM tumors [27,101,102]. The expression of family members p63 and p73 is tissue specific and can influence NCS self-renewal and differentiation. Therefore, downregulation of *TP63* and *TP73* may also contribute to the malignancy and treatment resistance of GBM [102,103]. Indeed, methylation of the promoter regions of *TP63* and *TP73* corresponded with higher grade GBM [104]. As GOF mut-p53 have been demonstrated to negatively regulate p63 and p73 activity, mut-p53 might be involved in the GSC’s resistance to differentiation and enhanced capacity for invasion [72,101]. GOF mut-p53’s status in GSC’s has largely been understudied and warrants further exploration. 

Despite a notable scarcity of literature focusing on GOF mut-p53 in GBM, a few recent studies have analyzed some of its pathological consequences. Ham et al. [105] demonstrated that GOF mut-p53 promotes inflammation in GBM. Bioinformatic analysis revealed that the ectopic expression of mut-p53 corresponded with the enhancement of inflammatory and chemotaxis genetic signatures. Specifically, upregulation of CCL2 and TNFA resulted in elevated levels of microglia and monocyte-derived immune cell infiltration. Additionally, the enhancement of these gene signatures predicted worse prognosis and shorter survival. Our group has shown that PTEN, another major tumor suppressor, displays aberrant tumor-promoting activity in GBM cell lines. This novel function is a result of PTEN regulating GOF mut-p53 expression through the inhibition of MDM2-mediated degradation and possibly also through direct protein interaction [106]. Moreover, PTEN, GOF mut-p53, CBP and NF-ɣ form a transcriptional complex in human GBM tumor samples. Molecular targets of this complex include the oncogenes c-Myc and Bcl-XL. Inhibition of mut-p53 with the small molecule PRIMA-1 exhibited greater antitumor effects in samples expressing PTEN, emphasizing their tumor promoting interaction [107]. 

GOF mut-p53 was previously reported to regulate chromatin-remodeling complexes (ex. SWI/SNF) to further control the expression of tumorigenic genes [108]. A study by Brazdova et al. [109] aiming to identify binding sites of mut-p53 demonstrated the reorganization of chromatin resulting in GOF mut-p53 binding to intronic and intergenic sequences. Analysis of U251 GBM cells demonstrated that GOF mut-p53 specifically binds G/C-rich DNA around transcription start sites associated with the histone mark H3K4me3 characterizing active chromatin marks [110]. Another recent study by Zhu et al. [111] examined the role of GOF mut-p53 DNA binding for identification of novel gene targets different from wt-p53. The analysis determined that GOF mut-p53 binds ETS2 for activation of chromatin remodeler genes such as MLL1, MLL2 (both H3K4 methyltransferases) and MOZ (an acetyltransferase). The examination of RNA expression in mut-p53 GBM tumors showed upregulation of MLL1, MLL2 and MOZ compared to *TP53* wild type or null tumors.

Consistent with observations made in other cancers, these few GBM studies exemplify the tumorigenic potential of GOF mut-p53. Previous GOF mut-p53 studies provide a useful framework for future research in GBM. Among other, the modes of action, functions and prognostic value of GOF mut-p53 in GBM warrant reevaluation.

## 3. P53-Targeted Therapies

**Figure 4:**
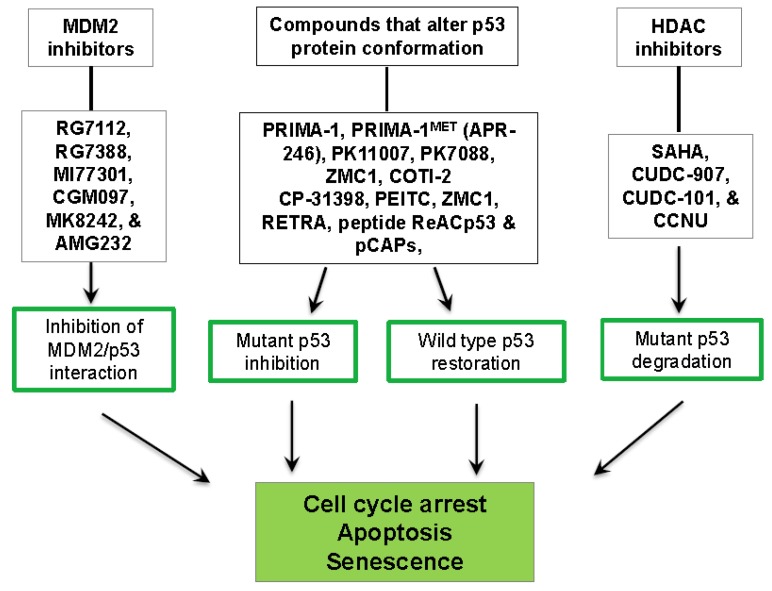
Summary of select strategies for the therapeutic targeting of mutant p53 in GBM. Approaches include restoration of wild type p53 activity or degradation of mutant p53.

The high prevalence and expression of p53 mutations in GBM makes them an important target for precision medicine therapies. Reactivating or restoring wt-p53 would be a promising therapy for many cancers. Various strategies have been explored to induce/reactivate wt-p53 or inhibit GOF mut-p53 in a wide range of cancer types, including GBM [113]. These strategies include inhibition of the MDM2/p53 complex to prevent wt-p53 degradation, restoration of wt-p53 function in mut-p53 tumors and inhibition of GOF mutant p53 (Figure 4).

### 3.1. Inhibition of the MDM2/p53 Complex

MDM2 is an E3 ubiquitin ligase that negatively regulates p53 by inducing its degradation in the proteasome. Inhibiting the MDM2/p53 interaction to reactivate p53 function is therefore a promising strategy for cancer and GBM therapy. Consequently, efforts to develop small-molecule inhibitors of MDM2/p53 interaction have been made and resulted in the discovery and testing of these inhibitors in cancer and GBM. Nutlins are such inhibitor molecules that were identified through chemical library screenings. The nutlin analog RG7112 was the first-in class MDM2 inhibitor [114]. Several other MDM2 inhibitors including RG7388, MI77301, CGM097, MK8242, and AMG232 were later developed and tested in clinical trials. Among these, AMG232 is the most potent MDM2 inhibitor described to date [115]. AMG232 effects were tested in the therapy resistant and putative tumor initiating GBM stem cells. AMG232 exhibited relative selectivity to wt-p53 stem cells was very efficacious in inhibiting three-dimensional tumor spheroids growth and stemness-related factors [116]. Another study found that nutlin3a-mediated inhibition of MDM2/p53 interaction and led to an impairment in DNA repair that correlated with potentiation of temozolomide-mediated cell death both in vitro and in vivo in GBM [45]. A preclinical evaluation of RG7112 MDM2 inhibitor across a panel of 36 patient-derived GBM cell lines genetically characterized according to their *TP53* pathway status was also performed. In MDM2-amplified cell lines, RG7112 restored p53 activity, crossed the blood–brain and the blood–tumor barrier and treatment of MDM2-amplified *TP53* wild-type xenografts with the inhibitor reduced tumor growth and increased animal survival [117]. Another study developed and tested in GBM novel indolylglyoxylyldipeptides that target both MDM2 and Translocator Protein (TSPO). The compounds bound TSPO and reactivated p53 functionality by dissociating it from MDM2. In GBM cells, these molecules caused dymdissipation and inhibition of cell viability, suggesting TSPO/MDM2 dual-targeting as a new anti-GBM therapy [118].

### 3.2. Restoration of wt-p53 Conformation and Function

GOF p53 mutations can be corrected by restoring wt-p53 function through introduction of additional point mutations that lead to p53 protein stabilization [112]. Current available compounds designed for rescuing wt-p53 function have been summarized in several review papers [119,120,121]. Most p53 alterations in GBM are point mutations that result in highly expressed mut-p53 protein and GOF. Consequently, efforts have been made to develop drugs that could reactivate wt-p53 by changing the conformation of the mut-p53 protein. A wide range of compounds have been developed for restoring wt-p53 function in different cancers, including GBM [119,120,121,122,123]. 

One of the most efficient molecules for p53 restoration, PRIMA-1 (2,2-bis(hydroxymethyl)-1-azabicyclo[2.2.2]octan-3-one), was discovered from a functional screening of the NCI drug library in cancer cells based on its ability to re-activate wt-p53 properties from select p53 missense mutants [124] (Figure 4). It has been widely studied and demonstrated to induce p21 expression, cell cycle arrest and apoptosis [124]. Mut-p53s are mostly not folded properly leading to unstable structure. PRIMA-1 was shown to alter mutant protein folding to restore wt-p53 conformation and p53 function [125]. PRIMA-1 inhibits cell growth and induces apoptosis in osteogenic Sarcoma Saos-2 cells [124] and primary human acute myeloid leukemia and chronic lymphoid leukemia cells [126,127]. In GBM, PRIMA-1^MET^ (APR-246) has been shown to inhibit of cell growth and stemness and induce apoptosis [128]. Importantly, PRIMA-1 and its structural analog PRIMA-1^MET^ (APR-246) inhibited tumor growth in mouse models of GBM [124,129,130]. PRIMA-1 has not been tested in patients with GBM. However, PRIMA-1^MET^ (APR-246) has been studied in a phase I/IIa clinical trial in patients with hematological malignancies and prostate cancer [131]. PRIMA-1 analog COTI-2 is being tested in a phase I trial in patients with advanced gynecological cancers [132].

Other compounds and peptides have also been developed to correct mut-p53 conformation and function in cancer. Small molecule CP-31398 stabilizes p53 conformation and promotes p53 activity in GBM cells and induces p21 expression in both wt-p53 and mut-p53 cells. CP-31398 treatment can induce cell death in caspase-independent and bcl-x(L)-insensitive manners [123]. Dietary compound PEITC can reactivate p53 mutants in vitro and in vivo and preferentially demonstrated growth-inhibitory activity in p53-R175H vs. p53-R273H and p53-R248Q tumor cells [133]. Small peptides with different molecular sizes and amino acid sequences have been designed to bind to different p53 regions to reactivate mut-p53 [132]. Peptide ReACp53 was described to prevent the amyloid-like aggregation of proteins with point mutations in p53-R248Q or p53-R175H. Treatment of ovarian cancer xenografts with ReACp53 resulted in decreased cell proliferation and tumor reduction [134]. Lead peptides, pCAPs, can restore proper p53 folding and activity and have been used to treat different mouse xenografts of different cancer types expressing mut-p53. The results showed tumor regression in colorectal, ovarian, and breast cancer [135]. For proper gene transcription, the p53 protein requires a large quantity of zinc to undergo correct folding and binding to DNA response units [136]. The thiosemicarbazone ZMC1 (NSC319726) targets zinc-binding to inhibit cell growth in mut-p53 R175H tumors [137]. These latter mut-p53 targeting compounds have not been tested in GBM to date.

### 3.3. Degradation of Mut-p53 

Another approach for p53-based therapies consists in enhancing protein turnover using HDAC inhibitors. Heat shock protein Hsp70 and Hsp90 play important roles in mut-p53 degradation. Mut-p53 binds to the Hsp70 and Hsp90 chaperone complex that requires an interaction with HDAC6 for proper functioning [67]. When HDAC6 is absent or inhibited, the complex is disrupted resulting the dissociation and degradation of the mut-p53 [67]. Several HDAC inhibitors, including CUDC-907, CCNU, CUDC-and vorinostat, have been tested in GBM cells and mouse models [138,139,140,141]. Singh and colleagues reported that an FDA-approved HDAC inhibitor vorinostat in combination with tranylcypromine, can reduce GBM stem cell viability in a GBM xenograft model and lead to changes in apoptosis-regulatory genes such as *TP53* and *TP73* [141]. HDAC inhibitor SAHA shows strong effects in destabilizing mut-p53 by dissociating HDAC6 from functional interaction with Hsp90 and thereby inhibits GBM growth and resistance to chemotherapy [142,143,144,145,146]. However, SAHA was recently found to also down regulate wt-p53 [147], suggesting that it should be applied only to homozygous mut-p53 tumors. CUDC-907, a dual inhibitor of HDAC and PI3K, can induce mut-p53 degradation and abolish NFκB- and FOXM1-mediated DNA damage response to radio-sensitize pediatric high-grade GBMs [138]. Furthermore, HDAC inhibitor CCNU has been shown to sensitize adult GBM to chemotherapy with lomustine via degradation of mut-p53 [139], while another HDAC inhibitor CUDC-101 has been found to enhance mut-p53 degradation leading to sensitization GBM cells to small molecule inhibitors of EGFR [140].

## 4. Controversies and Future Perspectives

P53 is a major tumor suppressor that is activated in response to stresses to prevent the propagation of damaged cells and maintain genomic stability by inducing cell cycle arrest and apoptosis in stressed cell. A number of controversies are associated with research on *TP53* in GBM. One area of contention is discerning the timeline of *TP53* mutation in tumor development. In secondary glioblastoma mutations of *TP53* might occur early along with IDH1 mutations [148]

The exact timing of *TP53* mutation in primary GBM tumors has not been discerned. The early mutation of *TP53* in secondary glioblastoma raises the question of whether *TP53* mutation is an initiating driver mutation. Another area of contention relates to the growing evidence that shows that not only loss of wt-p53 function, but GOF oncogenic mut-p53 can enhance cancer and GBM progression. This evidence is frequently not reflected in most mouse models of GBM that rely on knockout of the wt-p53 gene. Importantly also, despite its frequency of deregulation and its central role in cancer, attempts at exploiting the p53 pathway for cancer and GBM therapy have not been very successful to date. Future research is needed to fill out the gaps in knowledge especially on the effects and modes of action of GOF mut-p53 in cancer and GBM. Such research would profit from the use of more representative knock-in models of GOF mut-p53 instead of commonly used knockout models. Finally, the very high expression of GOF mut-p53 in human GBM represents a little exploited opportunity for the therapeutic targeting of mut-p53 tumors. Significant research and development are needed to achieve this therapeutic potential.
